# Antibiofilm and antibacterial effects of *Nigella sativa* extract against *Streptococcus mutans* predicting the role of fatty acids composition: *in vitro*, CLSM and *in* *silico* studies

**DOI:** 10.2340/biid.v13.45299

**Published:** 2026-01-28

**Authors:** Sherif ELsayed, Khaled EL-Adl, Doaa M. Sadony, Haidy A. Gad, Abdullah Yousef, Ahmed A. Radwan

**Affiliations:** a Biomaterials Department, Faculty of Dentistry, Galala University, Suez, Egypt; b Chemistry Department, Faculty of Pharmacy, Heliopolis University for Sustainable Development, Cairo, Egypt; c Pharmaceutical Medicinal Chemistry and Drug Design Department, Faculty of Pharmacy (Boys), Al-Azhar University, Nasr City, Cairo, Egypt; d Restorative and Dental Materials Department, National Research Centre, Dokki, Giza, Egypt; e Department of Pharmacognosy, Ain Shams University, Cairo, Egypt; f Basic & Medical Sciences Department, Faculty of Dentistry, Al-Ryada University for Science and Technology. Sadat City, Menoufia, Egypt; g Genetics and Cytology Department, National Research Centre (NRC), Cairo, Egypt

**Keywords:** *Nigella sativa*, Streptococcus mutans, oral biofilm, CLSM, fatty acids, MurA enzyme, molecular docking

## Abstract

**Purpose:**

To investigate the antibiofilm and antibacterial properties of *Nigella sativa* seed aqueous extract (NSE) against *Streptococcus (S.) mutans*.

**Methods:**

Chemical analysis of aqueous extract powder of *Nigella sativa* seeds was carried out using gas chromatography–mass spectrometry (GC-MS). *S. mutans* biofilm removal by NSE and chlorhexidine (CHX) mouthwash was evaluated using crystal violet (CV) assay. Ten extracted human teeth were sectioned to obtain 12 enamel specimens. Enamel specimens were coated by artificial saliva with bovine serum albumin, and then inoculated by *S. mutans* for biofilm formation. Enamel specimens were divided into four groups [positive biofilm control (without treatment), negative biofilm control (without bacteria), NSE and CHX biofilm treatment groups]. Acridine orange/propidium iodide assay was used for vital/dead biofilm staining. Confocal laser scanning microscopy (CLSM) biofilm images were digitally analyzed to calculate vital/dead stains relative fluorescence (RF) and estimate biofilm thicknesses. Molecular docking (MD) of interaction between long chain fatty acids (FAs) of NSE and MurA enzyme (PDB: 1UAE) was performed.

**Results:**

GC-MS revealed high concentration of long chain FAs (linoleic, oleic and palmitic acids). NSE and CHX groups showed significantly the highest red (dead) RF. NSE caused the highest reduction in biofilm thickness. NSE and CHX presented the minimum absorbance in CV assay. MD predicted a high binding affinity between FAs ligands and MurA receptors (Arg371, Arg331, Asn23, Lys22), with maximum affinity to linoleic acid (–98.07 kcal/mol).

**Conclusion:**

NSE showed antibacterial effect against *S. mutans* biofilm comparable to that of CHX, with a higher biofilm removal effect. NSE is expected to have a MurA enzyme inhibitory effect.

## Graphical abstract

**Figure:**
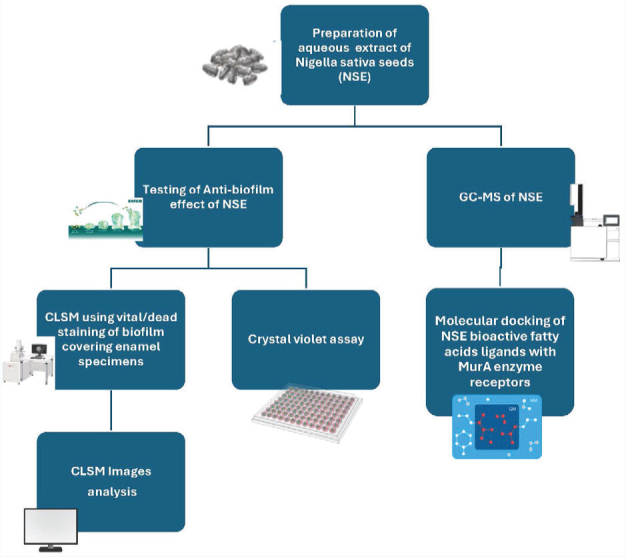
GRAPHICAL ABSTRACT

**KEY MESSAGES**

- Aqueous extract of *Nigella sativa* seeds (NSE) may have a biofilm removal effect against *Streptococcus mutans* higher than that of chlorhexidine mouthwash.
- NSE may also have antibacterial effect against *Streptococcus mutans* by inhibiting MurA enzyme.
- These findings may be attributed to the rich content of long chain fatty acids in NSE, especially linoleic acid.

## Introduction

The oral cavity is inhabited by multiple species of bacteria, which can grow under favorable conditions, such as moisture, acidic media and anaerobic conditions [1, 2]. *Streptococcus (S.) mutans* can synthesize a biofilm layer on the enamel surface of human teeth, giving signals to other species such as *S. sanguinis* to form stronger biofilm, leading to enamel demineralization and dental caries [3].

Chlorhexidine is a bis-biguanide chemical that was innovated in the 1940s and has been marketed as a disinfectant. After discovering its antiplaque activity, it was used as a mouthwash [4]. It is widely used as an adjunct therapy to mechanical debridement for controlling periodontal diseases [4]. The chemical structure of chlorhexidine is composed of a symmetric bis-biguanide molecule carrying two chloroguanide chains that are interconnected by a hexamethylene chain. Accordingly, chlorhexidine has two positive charges at physiological pH, acting as a strong base [5]. The antimicrobial mechanism of chlorhexidine is based on damaging the cytoplasmic membrane of bacteria by its positive charge [5].

Chlorhexidine mouthwash has been reported to have some side effects such as tooth staining, taste change, and mucosa irritation [6]. Reports of antibiotic resistance to chlorhexidine emerged recently [5, 7]. Recently, attention has also been drawn to the innovation of natural herbal mouthwash formulations, as a replacement for chlorhexidine [8].

*Nigella (N.) sativa* is a plant from the Ranunculaceae family that is present in large areas of Asia and Africa [9]. *N. sativa* seeds had been widely used as a traditional treatment for bacterial infections [10]. Secondary metabolites of *N. sativa* seeds such as flavonoids, terpenoids or alkaloids have antibacterial and antibiofilm effects against a wide range of bacterial species [11]. Several reports across the literature showed promising results of *N. sativa* seed extracts against oral pathogenic bacteria [12, 13].

To evaluate the effect of novel antibiofilm agents, quantification of biofilm development and viability following treatment is necessary. Using crystal violet (CV) stain in addition to spectrophotometry is one of the most widely used biofilm quantitative evaluation methods [14, 15]. However, traditional CV assay does not allow for 3D visualization and evaluation of bacterial biofilm [16].

Confocal laser scanning microscopy (CLSM) selectively excites a sample with fluorescence signals across different planes, to generate images point by point using laser excitation at specific wavelengths. CLSM has the advantage of enabling a 3D visualization of bacterial biofilm structure, by excluding signals from out of focus adjacent planes. Another advantage of CLSM is the ability to gain extra information using specific fluorescent stains added to the sample such as the presence of extracellular DNA or viability of biofilm [16].

Molecular docking (MD) helps in new drug discovery and in studying the biomechanisms of new bioactive compounds. This method is used to learn about the interaction between drug ligands and protein receptors. The orientation, energy and the nature of binding interaction between the ligand and the target protein receptor can be predicted, to study the performance and affinity of ligands [17].

In the current study, the *Nigella sativa* seed aqueous extract (NSE) was evaluated as an antibiofilm agent against *S. mutans* bacteria, in comparison to chlorhexidine (CHX) mouthwash. The correlation between the chemical composition of *N. sativa* seed extract and its antibiofilm and antibacterial actions was investigated. The first null hypothesis was that there was no difference between NSE and CHX, regarding the antibacterial action against *S. mutans* population in biofilm. The second null hypothesis was that there was no difference between NSE and CHX, regarding their *S. mutans* biofilm removal effect. A second aim of the present study was to explore a possible inhibitory mechanism of NSE against MurA enzyme of *S. mutans* using MD.

## Methods

### Biofilm establishment on enamel specimens

#### Preparation of enamel specimens

The study design, methods and the used materials were approved by the ethical committee of the Faculty of Oral and Dental medicine, Ahram Canadian University, Giza, Egypt., under license (IRB00012891#149). Ten extracted human teeth were obtained from the natural teeth bank at the National Research Centre, Giza, Egypt. They were used to prepare 12 enamel specimens. The teeth were disinfected using diluted sodium hypochlorite (1:10) for 10 minutes, then they were rinsed and cleaned by periodontal scalers and inspected visually to check for the absence of any defects or dental restorations. After inspection, the teeth were autoclaved. The sterilized teeth were stored in filtered water to keep them hydrated; the storage water was refreshed daily to prevent bacterial colonization.

The teeth were then sectioned at the cemento-enamel junction using the diamond disc of a Bronwill hard tissue microtome (Bronwill LBQ 094. VWR 77 machine) and water coolant. The buccal and lingual surfaces of the resulting crowns were used to obtain 12 rectangular enamel specimens (4–5 × 4–5 mm) with an approximate thickness of 0.5 mm.

The enamel specimens were treated very carefully to prevent the formation of cracks during manipulation. They were immersed in a 70% ethyl alcohol sonication bath for 30 minutes. After sonication, the enamel specimens were packed in sterilization pouches and steam-autoclaved for 30 minutes at temperature of 121°C.

#### Preparation of artificial saliva

Six hundred mL of distilled water was mixed with 0.625 g KCl, 0.059 g MgCl_2_.6H_2_O, 0.166 g CaCl_2._2H_2_O, 0.804 g K_2_HPO_4_, 0.326 g KH_2_PO_4_, and then 10 g of sodium carboxymethyl cellulose was added to the mixture, using a magnetic stirrer and a heating plate. The pH was adjusted to 7.15 by adding 0.5 NaOH using a pH meter. Carboxymethyl cellulose was added to increase the viscosity of the artificial saliva, thus acting as the mucin component of natural saliva, while the other components represented the inorganic portion of saliva [18]. The prepared mixture was autoclaved at 121°C for 30 minutes. Then, bovine serum albumin (BSA) was added to the sterile artificial saliva, with concentration of 50 ug/mL., to represent the protein component of the saliva. BSA was filtered by a 0.22 µm filter syringe, before addition to sterile artificial saliva [18].

#### Primary treatment of enamel specimens

The sterile enamel specimens of all groups were incubated in sterile BSA-treated artificial saliva for 24 hrs for the formation of artificial salivary protein pellicle on the enamel specimens [19].

#### Biofilm formation on enamel specimens

Sterile brain heart infusion (BHI) broth media supplemented with 2% sucrose [20] was used to prepare an inoculum of *Streptococcus mutans* (ATCC 25175), starting with 0.5 MacFarland concentration of about 1.5 × 10^8^ CFU/mL. The inoculum was incubated under anaerobic conditions at temperature of 37°C for 48 hrs [21]. After incubation, diluted preparations of inoculum (1/1000 = 1.5 × 10^5–6^ CFU/mL) were transferred to glass test tubes.

Each primary treated enamel specimen (coated with artificial salivary pellicle) was submerged in the diluted inoculum individually inside a test tube. The test tubes were tightly closed and incubated under anaerobic conditions, at 37°C for 72 hrs, to ensure complete formation of biofilm [21]. The pellicle-coated (primary treated) enamel specimens of the negative biofilm control group were incubated inside glass tubes, filled with sterile BHI broth media only (without bacterial inoculation), under the same incubation conditions of the other groups.

#### Grouping of enamel specimens

The enamel specimens were divided into four groups according to the treatment protocol as follows:

**Positive biofilm control group:** The primary treated enamel specimens were incubated for *S. mutans* biofilm formation, without any treatment with antibiofilm agent (*n* = 2).

**Negative biofilm control group:** The primary treated enamel specimens were incubated in sterile BHI broth without any bacterial inoculation (i.e. under sterile conditions) (*n* = 2).

**Chlorhexidine hydrochloride mouthwash (CHX) group:** The primary treated enamel specimens were incubated for *S. mutans* biofilm formation, and after 72 hrs, the biofilm-coated enamel specimens were treated with chlorhexidine hydrochloride (125 mg/100 mL) mouthwash (CHX) (Hexitol, ADCO pharmaceuticals, El Amireya, Cairo, Egypt) (*n* = 3).

**NSE group:** The primary treated enamel specimens were incubated for *S. mutans* biofilm formation, then after 72 hrs, the biofilm-coated enamel specimens were treated with NSE preparation (*n* = 3).

### Preparation of aqueous extract of Nigella sativa seeds

One hundred grams of *N. sativa* seeds (Imtenan, Obour city, Cairo, Egypt.) were ground and subsequently boiled in one liter of distilled water for a duration of 20 minutes. The resulting mixture was carefully filtered first by sterile cotton piece to eliminate gross particles, followed by finer filtration through Whatman® qualitative filter paper No.1. After the filtration process, the water solvent of the aqueous mixture was eliminated by evaporation using rotary evaporator (rotavap). The resultant crude *N. sativa* seed extract was carefully collected from the rotary flask by dissolving it in small quantities of water, followed by drying in a desiccator. After desiccation, the dried aqueous extract seeds powder was stored at –20 °C. NSE test preparation was made by dissolving the dried aqueous extract seed powder in deionized water, using a seed powder to water ratio of 1:3 w/v.

### Gas chromatography–mass spectrometry analysis

The analysis of the chemical composition of the aqueous extract powder of *N. sativa* seeds was performed using GC-TSQ mass spectrometer (Thermo Scientific, Austin, TX, USA) supported by direct capillary column TG–5MS (30 m × 0.25 mm × 0.25 µm film thickness). The column oven temperature was held at 60°C at the beginning, and the temperature was increased by a rate of 5°C/min to 250°C, with a holding time of 2 minutes. The temperature was increased again to 300°C by a rate of 30°C/min. The injector temperature was kept at 270°C. The carrier gas was helium with a constant flow rate of 1 mL/min. The components were detected by comparing their mass spectra with those of WILEY 09 and NIST14 database [22].

### Anti-biofilm activity evaluation

#### CV assay

Diluted inoculum of *S. mutans* (ATCC 25175) (1.5 × 10^5–6^ CFU/mL) in BHI broth was prepared as described above. From the diluted bacterial culture, 300 µL volume was added to each well of three rows in a 96-well plate (five wells for each row). Also, 300 µL of sterile BHI broth without bacteria was added to the five wells of the fourth row in the 96-well plate [20]. The plate was incubated under anaerobic conditions at 37°C for 4 days for complete formation of biofilm. Then, the plate was inverted and the wells were washed three times by sterile phosphate buffer solution (PBS) to remove non-adherent cells [20].

Subsequently, 200 µL of the NSE and CHX antibiofilm preparations were added to two rows of washed wells (a row for each treatment group preparation). The plate was then incubated at 37°C for 10 minutes inside a shaker (100 rpm), then inverted, and the wells were washed with sterile PBS three times. This was followed by a methanol fixation treatment (200 µL) for each well. Subsequently, 200 µL of CV (0.1%) staining was added to each well for 15 minutes followed by three times washing using sterile PBS, and drying in an oven at 55°C. Finally, 200 µL of 33% glacial acetic acid was added to each well to resolubilize the CV. Absorbance was measured at 630 nm using a spectrophotometer microplate reader [20].

The row of wells with diluted bacterial culture without antibiofilm treatment represented the positive biofilm control group (control), while the row of wells filled with sterile BHI media represented the negative biofilm control group (blank).

The equation of biofilm removal percentage was calculated as follows [23]:


$$\text{The mean absorbance of}\frac{\left(\left(control-blank\right)-\left(treatment-blank\right)\right)}{\left(control-blank\right)}*100$$

#### Confocal laser fluorescence scanning microscope imaging

CLSM (Leica TCS SP8 CLMS equipped with LAS × 3.5.5.19976 software platform; Leica, Wetzlar, Germany) was used for scanning all enamel specimens.

Before scanning and after the biofilm incubation period of the enamel specimens of all groups, the broth was aspirated from each tube, and the enamel specimens were transferred gently to other separate glass tubes (one for each specimen) filled with sterile PBS, to remove planktonic bacteria by gentle aspiration and refreshing of sterile PBS using sterile pipette tips. Next, the enamel specimens of both CHX and NSE groups were incubated separately in test tubes (one for each enamel specimen) filled with the assigned antibiofilm agent (according to the designated group), and then incubated in a shaker (100 rpm) with a temperature of 37°C for about 10 minutes. After treatment, the antibiofilm agents were removed by gentle aspiration and replaced by sterile PBS in each test tube, and the PBS was refreshed about three times in each tube.

Enamel specimens of all groups were scanned using acridine orange/propidium iodide (AO/PI) dual staining to evaluate the vitality of bacterial cells. The vital bacteria stained by AO displayed green fluorescence and the dead bacteria stained by PI emitted red fluorescence [24].

Laser lines were adjusted at 488/552 nm wavelengths for excitation. Detector lines for emission wavelength for channel 1 (green) were adjusted at 493 nm – 547 nm, and for channel 2 (red) were adjusted at 600 nm – 727 nm.

#### CLSM image analysis

Analysis of the CLSM images of vital/dead stain was carried out using ImageJ (1.48v, NIH, USA), which is open source and supported by several plugins for different functions of image analysis.

#### Quantitative analysis of relative fluorescence

Two different stack images from two different locations of each enamel specimens were analyzed for both green (vital) and red (dead) relative fluorescence (RF), with a total of six stack images for each of NSE and CHX groups and four stack images for each of negative and positive biofilm control groups.

Each stack image was split into two channels (green and red). The standard deviation projection of the *Z*-series of each channel stack image was generated using the *Z*-project function, accessed using the algorithm of image>Stacks>Z Project.

The resultant image was analyzed to calculate the fluorescence of each channel Z-projection image, by calculating mean gray value, using Analyze>measure software function.

RF of each channel was calculated by dividing the mean gray value of each channel *Z*-projection by the mean gray value of the *Z*-projection of the original stack image before splitting.


$$\text{RF}=\frac{\text{mean gray value of each channel Z projection}}{\text{mean gray value of original stack image Z projection}}$$

#### Quantitative biofilm thickness measurements

Two different *Z*-stack images from each enamel specimen were analyzed for the biofilm cross section thickness measurement, using the ImageJ Analyze function, with a total of 6 *Z*-stack images for each of NSE and CHX and 3 *Z*-stack images for the positive biofilm control group. The images used were 3D *Z*-stack images generated by the LAS × 3.5.5.19976 software platform. Seven thickness measurements were taken from different locations of the fluorescent region (FR) cross section in each *Z*-stack image. The FR thickness gave indication about the biofilm thickness, but it did not represent the actual thickness of biofilm, due to the autofluorescence of enamel [25]. Thus, ‘fluorescent region (FR) thickness’ would be a more accurate expression, than ‘biofilm thickness’.

### Molecular docking

Molsoft software (Molsoft L.L.C., San Diego, USA) was applied to simulate the molecular binding between the three most prominent fatty acids (FAs) in NSE according to gas chromatography–mass spectrometry (GC-MS) analysis (linoleic acid, oleic acid [OA] and palmitic acids), and MurA enzyme of *S. mutans* [17]. Molsoft permits flexible ligand docking as it is an integrated suite of automated docking tools. It predicts the type of binding between drug candidates or substrates (small molecules) and a receptor of known 3D structure. The protein target 3D structure needed to be prepared and modeled according to the format requirements of the docking algorithms used. Therefore, the crystal structure of the receptor was downloaded from the Brookhaven Protein Databank (PDB: 1UAE) [17] using Molsoft program. The protein was prepared for MD by the addition of polar hydrogens into the protein atoms. The protein active site was defined by placing a grid over the center of co-crystallized ligand. All the necessary grid maps were calculated before a protein was ready for docking simulations. Ligand and protein interactions produced docked energy which was calculated as an interaction energy [26, 27].

### Statistical analysis

Statistical analysis of the results of the CV assay and results of the CLSM image analysis (RF & FR thickness) was performed with SPSS 27, Graph Pad Prism, and Microsoft Excel 2016. All data was presented as means and standard deviations. All data was explored for normality by using Shapiro Wilk and Kolmogorov-Smirnov normality tests, which revealed that all data was normally distributed. Accordingly, comparison between groups was performed by using One Way Analysis of variance (ANOVA) test followed by Tukey Post Hoc test, while comparison between red and green RF was performed by using Paired *t*-tests. For qualitative data, all comparisons were performed by using Fishers Exact tests. The significant level was set at *p* ≤ 0.05.

## Results

### GC-MS analysis

The results of GC-MS analysis are presented in Table 1 and revealed high concentrations of glycerol and long chain FAs (linoleic, oleic and palmitic acids).

**Table 1:** Results of GC-MS analysis.

Chemical compound	Molecular formula	Molecular weight	CAS number	Area percentage (%)	Retention time (minute)
1,2,3-Butanetriol, 3TMS	C_13_H_34_O_3_Si_3_	322.6638	33581–76–9	6.59	10.1
Glycerol, 3TMS	C_12_H_32_O_3_Si_3_	308.6372	6787–10–6	18.1	12.7
Palmitic acid, methyl ester	C_17_H_34_O_2_	270.4507	112–39–0	6.9	27.45
Palmitic Acid, TMS	C_19_H_40_O_2_Si	328.6052	55520–89–3	17.05	30.11
Oleic acid, methyl ester	C_19_H_36_O_2_	296.4879	112–62–9	0.39	30.75
Linoleic acid	C_18_H_32_O_2_	280.4455	60–33–3	13.47	31.9
Oleic acid	C_18_H_34_O_2_	282.4614	112–80-1w	3.67	32
Linoleic acid TMS	C_21_H_40_O_2_Si	352.6266	56259–07–5	14.35	33.01
Oleic Acid, (Z)-, TMS	C_21_H_42_O_2_Si	354.6425	21556–26–3	7.18	33.13
2-Oleoylglycerol, 2TMS	C_27_H_56_O_4_Si_2_	500.9021	56554–42–8	3.37	40.77
Linolenic acid	C_18_H_30_O_2_	278.4296	463–40–1	1.8	41.15
1,3-Butanediol, 2TMS	C_10_H_26_O_2_Si_2_	234.4832	56771–47–2	2.3	9.72

GC-MS: gas chromatography–mass spectrometry; TMS: Trimethylsilyl derivatives; CAS: Chemical Abstracts Service

### Determination of biofilm removal activity by CV assay

**Figure 1:**
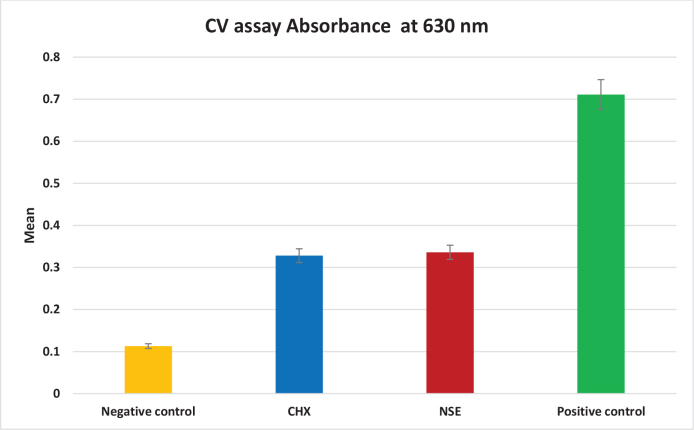
Bar chart showing CV absorbance in all groups. CV: crystal violet.

The absorbance of CV staining in the 96 plate is presented in Table 2 and Figure 1. The One-Way ANOVA test revealed a significant difference between groups (*p* < 0.0001), indicating varying levels of remaining biofilm biomass after different treatments. The negative biofilm control (no bacterial inoculation) group exhibited the significantly lowest absorbance reading (0.113 ± 0.006), while the positive biofilm control (no treatment) showed the significantly highest (0.711 ± 0.133) absorbance reading. Both CHX and NSE recorded intermediate absorbance values (0.328 ± 0.040 and 0.336 ± 0.075, respectively), with no significant difference between CHX and NSE.

**Table 2:** Mean and standard deviation of absorbance reading at 630 nm in all groups of CV assay.

Absorbance at 630 nm	Mean	Std. Deviation	*P* value
Blank (negative biofilm control)	0.113^a^	0.006	< 0.0001*
CHX	0.328^b^	0.040	
NSE	0.336^b^	0.075	
Positive biofilm control	0.711^c^	0.133	

* Significant difference as *p* ≤ 0.05.

Means with different superscript letters per column were significantly different as *p* < 0.05. CV: crystal violet; CHX: chlorhexidine; NSE: *Nigella sativa* seeds aqueous extract.

**Figure 2:**
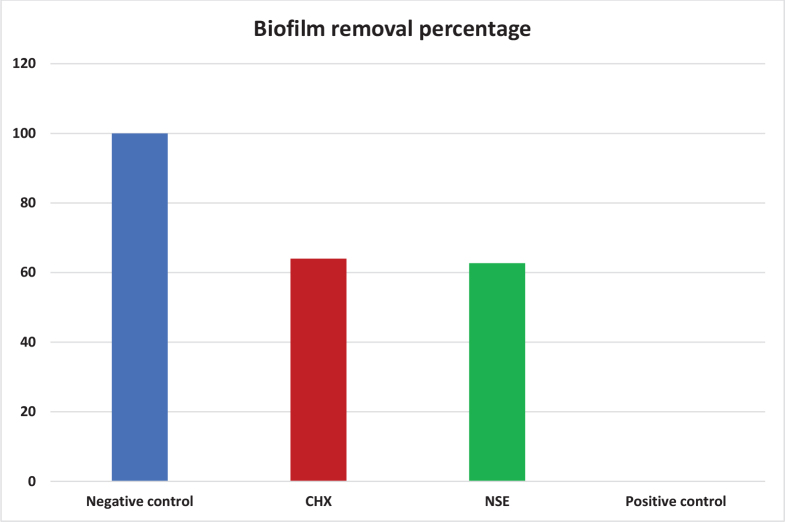
Percentage of biofilm removal in CV assay of all groups. CV: crystal violet.

The biofilm removal percentage evaluated by CV assay is presented in Table 3 and Figure 2. The Fisher’s Exact test found the percentage to differ significantly (*p* < 0.0001) between control groups (the negative and positive biofilm control groups) and treatment groups (CHX and NSE). CHX achieved 64%, followed by NSE (62.7%) with statistically similar percentages.

**Table 3:** Percentage of biofilm removal in CV assay of all groups.

Biofilm removal percentage	%	*P* value
Blank (negative biofilm control)	100^a^	< 0.0001*
CHX	64^b^	
NSE	62.7^b^	
Positive biofilm control	0^c^	

* Significant difference as *p* ≤ 0.05.

Means with different superscript letters per column were significantly different as *p* < 0.05. CV: crystal violet; CHX: chlorhexidine; NSE: *Nigella sativa* seeds aqueous extract.

### Analysis of RF in CLSM images

**Figure 3:**
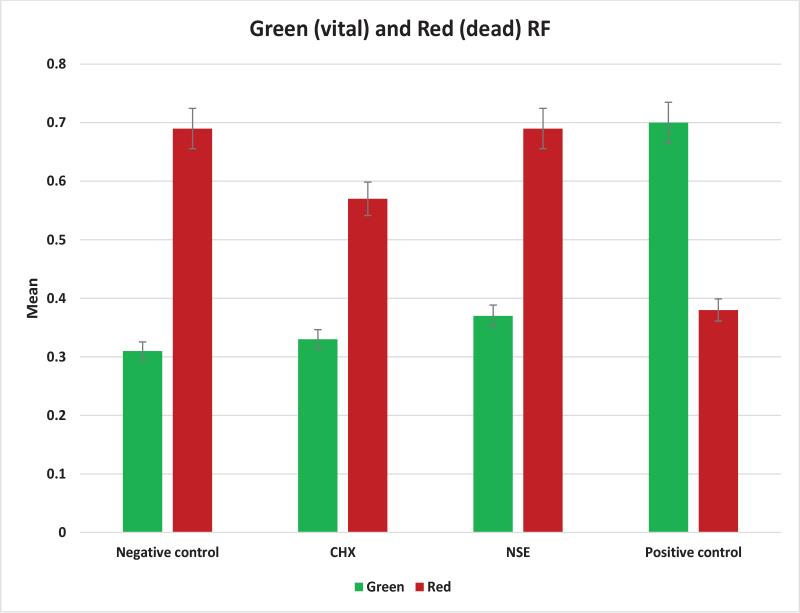
Bar chart showing red and green RF in all groups. RF: relative fluorescence.

CLSM RF of green (vital) and red (dead) staining, in *S. mutans* biofilm that had formed on the enamel specimens of the four groups, was assessed and presented in Table 4 and Figure 3.

**Table 4:** Mean and standard deviation of green and red RF in S. mutans biofilm.

Dental biofilm (*S. mutans*)	Green RF		Red RF		Mean difference	Std. error difference	95% confidence interval of the difference		*P* valuePaired *t* test
	Mean	Std. deviation	Mean	Std. deviation			Lower	Upper	
Negative control	0.31^a^	0.03	0.69^a^	0.02	–0.38	0.02	–0.43	–0.34	< 0.0001*
CHX	0.33^a^	0.03	0.57^a^	0.06	–0.25	0.03	–0.31	–0.19	< 0.0001*
NSE	0.37^a^	0.10	0.69^a^	0.10	–0.32	0.06	–0.45	–0.19	< 0.0001*
Positive control	0.70^b^	0.09	0.38^b^	0.05	0.32	0.05	0.19	0.44	< 0.0001*
*P* value One Way ANOVA test	< 0.0001*		< 0.0001*						

* Significant difference as *p* ≤ 0.05.

Means with different superscript letters per column were significantly different as *p* < 0.05. RF: relative fluorescence; CHX: chlorhexidine; NSE: *Nigella sativa* seeds aqueous extract.

#### Intergroup comparison (Comparison between groups)

The One-Way ANOVA test revealed statistically significant differences in the green and the red RF among the four tested groups (*p* < 0.0001). According to the post hoc test of green RF, the negative biofilm control (no bacterial inoculation), CHX, and NSE groups had significantly lower and statistically similar fluorescence compared to the positive biofilm control group (without treatment), with positive biofilm control group presenting the highest bacterial vitality among groups [16, 21].

**Figure 4:**
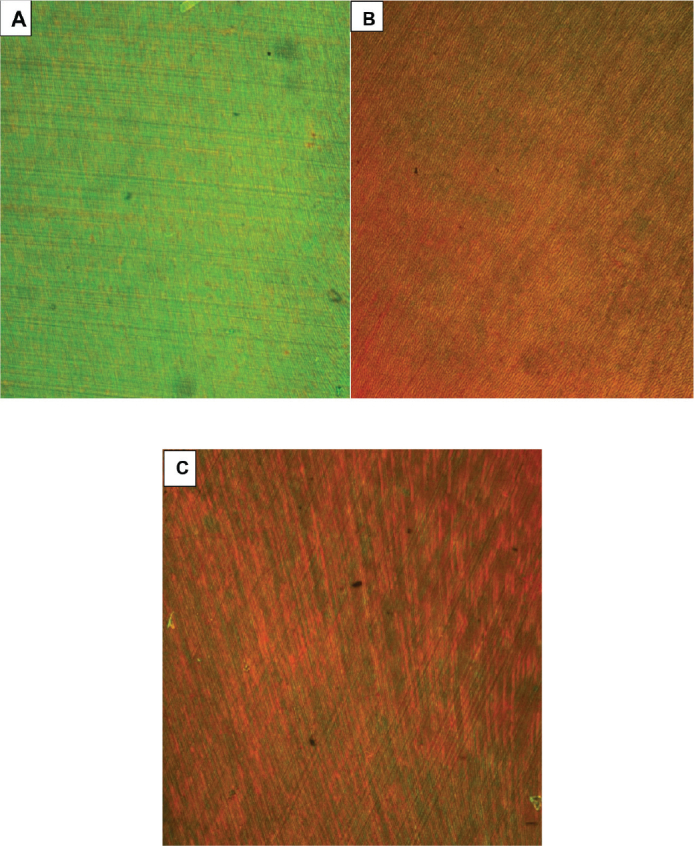
Z-projection standard deviation summation of CLSM merged stack images of S. mutans biofilm stained by AO/PI assay: (A) positive biofilm control group, (B) CHX group, (C) NSE group. CLSM: Confocal laser scanning microscopy; AO/PI: acridine orange/propidium iodide; CHX: chlorhexidine; NSE: *Nigella sativa* seed aqueous extract.

Regarding red RF, the reverse effect was observed, as the negative biofilm control, CHX, and NSE groups showed significantly higher red RF mean values and statistically similar fluorescence than the positive biofilm control group (without treatment), presenting the least bacterial vitality among groups (Figure 4).

#### Intragroup comparisons

The paired *t*-test conducted to compare between red and green RF within each group demonstrated a highly significant difference between green and red RF mean values within all groups (*p* < 0.0001). The mean differences ranged from –0.25 to –0.38 in negative biofilm control, CHX, and NSE groups, indicating higher values in the red RF compared to the green RF scores. Conversely, the positive biofilm control group exhibited a mean difference of +0.32, reflecting higher values in green RF than in red RF.

### Analysis of FR thickness in Z-stack CLSM images

**Figure 5:**
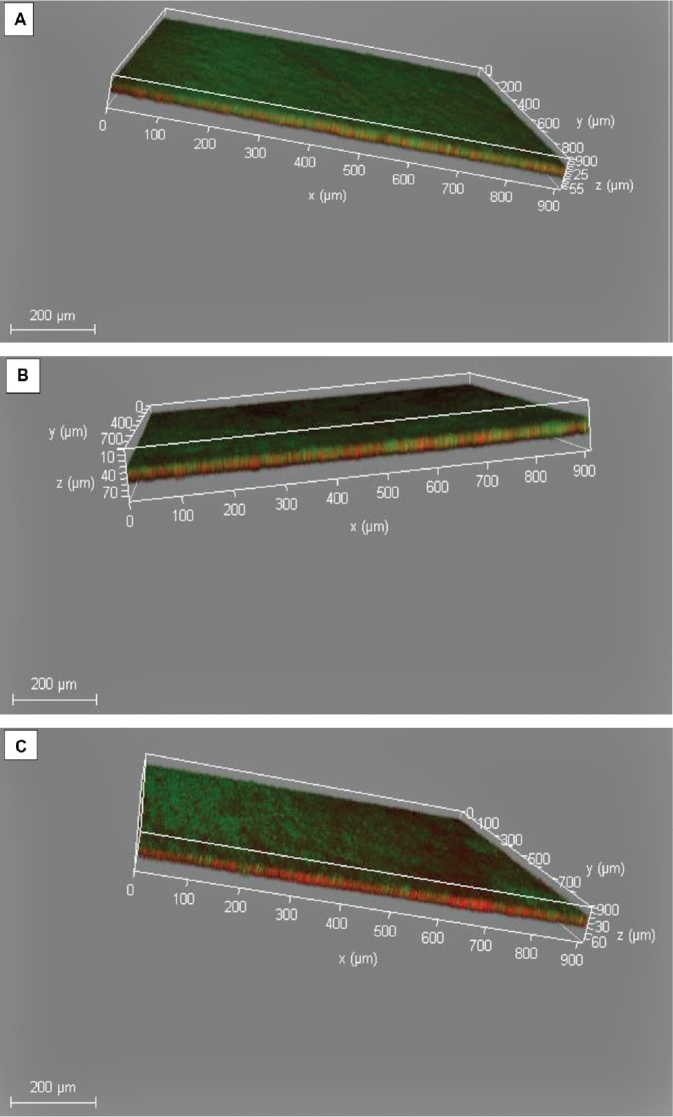
Z- stack CLSM images of S. mutans biofilm formed on human enamel specimens: (A) positive biofilm control group, (B) CHX group, (C) NSE group. CLSM: Confocal laser scanning microscopy; CHX: chlorhexidine; NSE: *Nigella sativa* seeds aqueous extract.

**Figure 6:**
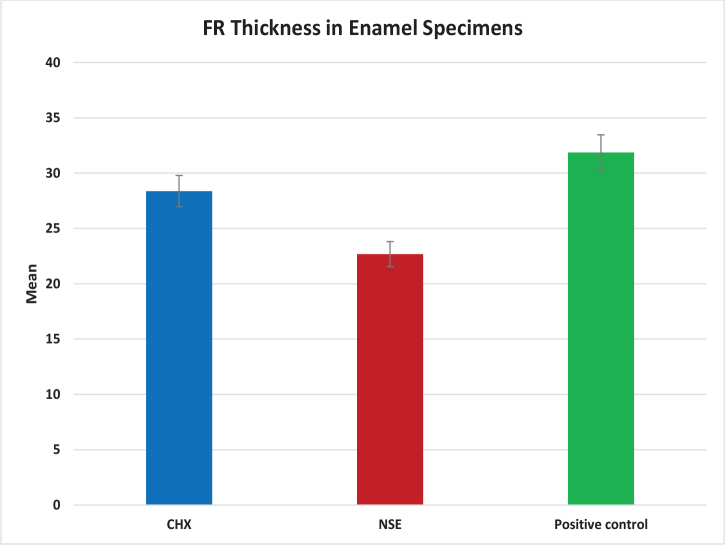
Bar chart showing thickness of fluorescent region of enamel specimens in CHX, NSE, and positive control groups according to Z-stack CLSM image analysis. CLSM: Confocal laser scanning microscopy; CHX: chlorhexidine; NSE: *Nigella sativa* seeds aqueous extract.

The Z-stack CLSM images generated by **LAS** × **3.5.5.19976 software platform** were subjected to biofilm thickness analysis by ImageJ software (Figure 5). The results of the FR thickness measurements are presented in Table 5 and Figure 6, for CHX, NSE and positive biofilm control groups. The One-Way ANOVA test revealed a significant difference between the three groups (*p* < 0.05), and the Tukey’s Post Hoc test found that the positive biofilm control group exhibited significantly the highest mean FR thickness, followed by the CHX group, while the NSE group showed significantly the lowest FR thickness.

**Table 5:** Mean and standard deviation for thickness of FR in enamel specimens (µm) of CHX, NSE, and positive biofilm control groups.

Thickness	Mean (µm)	Std. deviation	*P* value
CHX	28.37^b^	4.905	< 0.0001*
NSE	22.68^a^	3.743	
Positive biofilm control	31.87^c^	3.513	

* Significant difference as *p* ≤ 0.05.

Means with different superscript letters per column were significantly different as *p* < 0.05. CHX: chlorhexidine; NSE: *Nigella sativa* seed aqueous extract; FR: fluorescent region.

### Docking results

**Figure 7:**
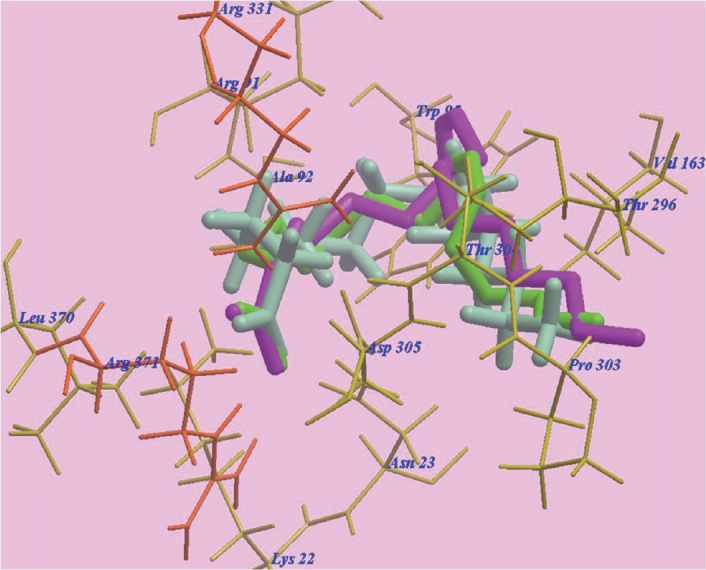
Superimposition of Linoleic acid, Oleic acid and Palmitic acid in the active sites of MurA enzyme (PDB: 1UAE).

**Figure 8:**
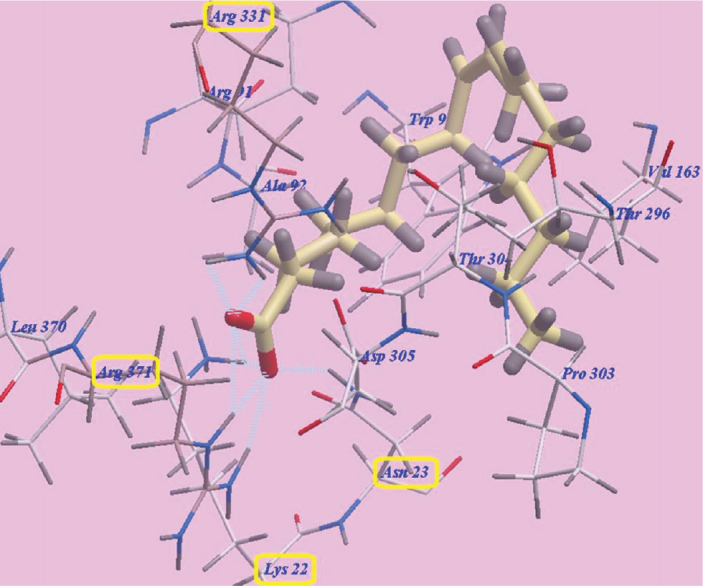
Binding orientation of Linoleic acid in the active site of MurA enzyme (PDB: 1UAE).

The three FAs, namely linoleic acid, OA and palmitic acid exhibited nearly the same binding orientation inside the active site of MurA enzyme (Figure 7). Linoleic acid exhibited the highest binding affinity towards MurA enzyme (PDB: 1UAE) with -98.07 kcal/mol with the formation of 8 H-bonds with Arg371 (2.07 Å, 2.51 Å and 2.96 Å), Arg331 (2.45 Å and 2.95 Å), Asn23 (2.06 Å) and Lys22 (2.95 Å and 2.98 Å). The remaining aliphatic long chain exhibited hydrophobic interactions with amino acid residues present in the hydrophobic pockets around them in the receptor (Figure 8).

**Figure 9:**
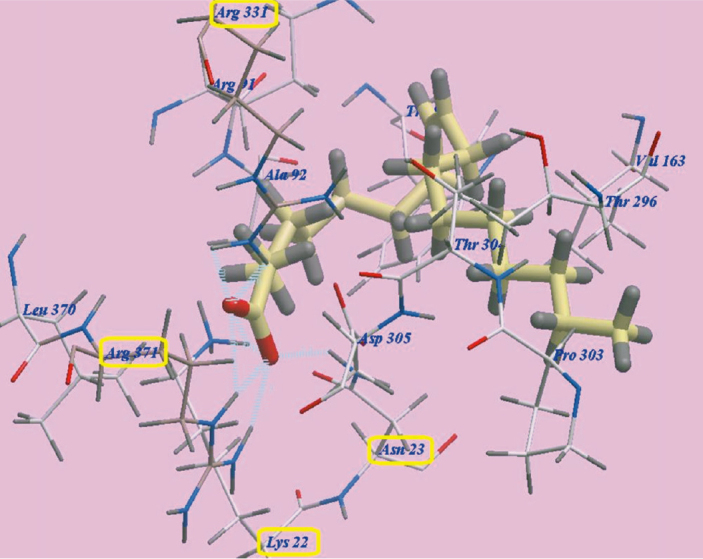
Binding orientation of Oleic acid in the active site of MurA enzyme (PDB: 1UAE).

Also, OA displayed high binding affinity towards MurA enzyme (PDB: 1UAE) with -95.52 kcal/mol with the formation of 8 H-bonds with Arg371 (1.90 Å, 2.42 Å and 2.79 Å), Arg331 (2.55 Å and 2.97 Å), Asn23 (2.16 Å) and Lys22 (2.97 Å and 2.99 Å). The remaining aliphatic long chain exhibited hydrophobic interactions with amino acid residues present in the hydrophobic pockets around them in the receptor (Figure 9).

**Figure 10:**
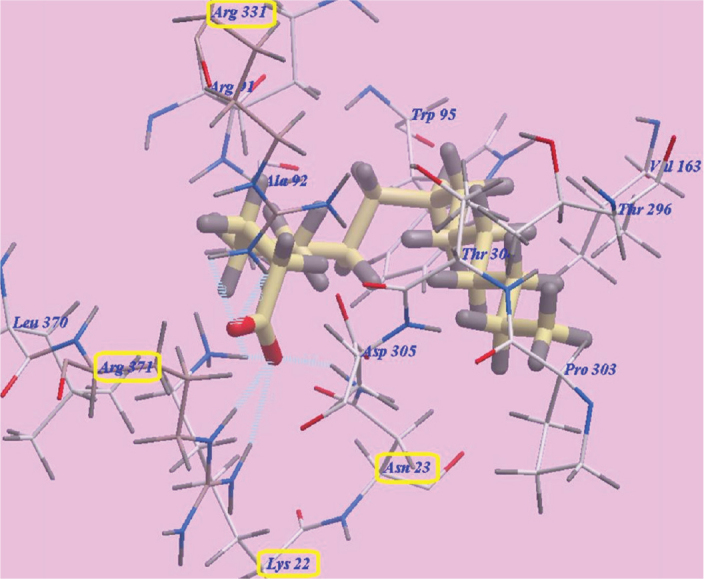
Binding orientation of Palmitic acid in the active sites of MurA enzyme (PDB: 1UAE).

Moreover, palmitic acid also presented high binding affinity towards MurA enzyme (PDB: 1UAE) with -90.96 kcal/mol with the formation of 8 H-bonds with Arg371 (2.08 Å, 2.27 Å and 2.97 Å), Arg331 (2.46 Å and 2.98 Å), Asn23 (1.99 Å) and Lys22 (2.98 Å and 2.99 Å). The remaining aliphatic long chain exhibited hydrophobic interactions with amino acid residues present in the hydrophobic pockets around them in the receptor (Figure 10). The data obtained from MD confirmed that the three FAs in NSE exhibited high affinities towards the MurA enzyme. Consequently, they have the potential to inhibit the cell wall synthesis of *S. mutans*.

## Discussion

In the current study, the antibiofilm effect of NSE against *S. mutans* biofilm was evaluated and compared to that of the widely used chlorhexidine mouthwash. The *in vitro* biofilm evaluation methods in this study included both CV assay and CLSM using a staining method. CLSM has several advantages, such as in situ, real-time, and 3D nondestructive observation of living biofilms [28]. However, the results of CLSM are generally affected by many factors, such as the concentration of stain, the proportions of live/dead bacteria, and staining quality, method and conditions. Also, it does not give accurate results in the case of multiple-species biofilms [29–31]. Accordingly, a single-species biofilm evaluation was conducted in this study, and more than one method was used to study and validate the biofilm formation and removal (CV assay and CLSM of vital/dead staining of biofilm) which gave more assurance and confirmation of results [16, 20].

Another consideration that was taken into account in this study was the accuracy of the terminology used to describe the bacterial state. As the staining method cannot confirm the viability of bacterial cells (i.e. ability of bacteria to grow), the terms ‘vital/dead’ were used in this study instead of the terms ‘viable/dead’ [29]. Overestimation of dead bacterial cells could occur due to staining of the extracellular DNA in the biofilm matrix by propidium iodide (PI) ‘red stain’ [16, 32]. Accordingly, including positive biofilm control group in this study as a baseline for the calculation of red stain RF intensity was crucial to avoid overestimation of the antibacterial effect of the testing agents [32].

Research studies in literature on CLSM image analysis are divided into two types: qualitative analysis studies which depend on simple subjective visualization of biofilm image color, or quantitative analysis studies which depend on the calculation of color intensity. However, most of the quantitative image analysis studies reported in literature were not explained in detail, regarding the type of the software algorithm followed and the segmentation method used [16, 24, 33, 34]. Accordingly in the current study, a quantitative analysis of RF color intensities was the preferred method to avoid subjectivity of the qualitative method. Also, the sequence of steps of image analysis using Imagej software were demonstrated in detail, for the sake of reproducibility and comparability across literature [16].

The protocol followed in the current study, for example, the primary treatment of enamel specimens using artificial saliva with BSA [18], was in accordance with the ISO standards for biofilm-biomaterial interface testing [19, 35]. As the salivary pellicle is initially formed within minutes on enamel surface intraorally, before attachment of bacteria and formation of biofilm, this pellicle provides the necessary protein attachments for the invading oral bacteria. Therefore in the present study, the treatment of the human enamel specimens with artificial salivary protein pellicle was crucial, to accurately replicate the process of biofilm formation inside the oral cavity [19, 36].

NSE showed comparable values to CHX regarding both *S. mutans* biofilm removal percentage, which was tested quantitatively by CV assay [37], and antibacterial effect tested by RF calculations of green and red staining color intensities (vital and dead bacteria). These results are in accordance with the results of other studies, that confirmed the antibacterial and antibiofilm effect of *N. sativa* seed extract against oral pathogens [11]. Thymoquinone (TQ) is one of the most potent bioactive components of *N. sativa* seed extract. In their study, Alamoudi et al. demonstrated the excellent antibacterial effect of TQ against root canal bacteria (*Streptococcus sanguis, Enterococcus faecalis, Prevotella intermedia, and Porphyromonas gingivalis*), in comparison to sodium hypochlorite [38]. Another study which evaluated the effect of TQ against four oral pathogens in comparison to chlorhexidine found that *S. mutans*, and *Staphylococcus aureus* showed larger inhibition zones with TQ than those with chlorhexidine [39].

By reviewing the literature about the antibacterial and antibiofilm effect of *N. sativa* seed extract against oral bacteria, it was observed that most of the studies concentrated only on the effect of TQ. Only limited research studies investigated the effect of the whole extract composition of *N. sativa* seeds against oral pathogens [40, 41]. *N. sativa* seed extracts are rich in several bioactive compounds other than TQ such as polyphenols, tannins, FAs or saponin [40, 42], which have antibacterial, antifungal and anti-inflammatory properties [11]. The *N. sativa* seed extract composition is highly affected by the origin of seeds, method of extraction and the type of the solvent used [43–46].

The GC-MS analysis of the aqueous extract of *N. sativa* seeds in the current study revealed high concentrations of long chain FAs which were unsaturated (linoleic and OAs) and saturated (palmitic acid). The analysis result is in accordance with the results of other studies that revealed the rich content of unsaturated and saturated FAs and triacylglycerols in *N. sativa* seed extract [44, 46]. The polarity of the solvent used for extraction has a significant effect on the composition and percentage of the FAs in the *N. sativa* seed extracts [44, 46]. The high polarity of water solvent used in the current study for extraction could explain the rich content of FAs in the aqueous extract of *N. sativa* seeds [46].

According to the results of vital/dead staining in the current study, NSE application for about 10 minutes significantly increased the red RF intensity, in comparison to the positive biofilm control group, which indicated an increase in the *S. mutans* cell membrane permeability or perforations, leading to an increase in PI stain uptake [47], which could be explained by the rich content of FAs in NSE. The antibacterial effect of FAs is well documented in literature [47, 48]. FAs have several antibacterial mechanisms depending on their chemical composition and the target bacterial population [48]. Chemical composition varieties such as the presence and the number of double or triple bonds, the length of the aliphatic chain and presence of ring moieties could have a significant effect on the antibacterial properties of FAs [48, 49].

Several studies have investigated the specific antibacterial and antibiofilm effect of FAs on *S. mutans* [49–51]. In the present study, the most prominent FAs presented in GC-MS analysis of *N. sativa* seeds aqueous extract were linoleic acid, palmitic acid and OA. Dilika et al. demonstrated that linoleic acid had antibacterial effect against *S. mutans* at minimum inhibitory concentration (MIC) of 12.5 µg/mL [49]. Linoleic acid (LA) which is a double bond polyunsaturated long chain (18 C) FA [52], showed ability to alter the synthesis of peptidoglycans (PG) in *Staphylococcus aureus*. Also, LA displayed inhibition action against FabI, which is an essential protein in FA synthesis of some types of bacteria; LA may exert antibacterial action by altering the structure and causing the destruction of bacterial cell wall [48].

Chamlagain et al. illustrated another antibacterial mechanism of polyunsaturated FAs against *S. mutans*. By investigating the antibiofilm effect of arachidonic fatty acid (AA) against *S. mutans*, they concluded that AAs can target the bacterial cell membrane by their incorporation in the cell membrane of the bacterial cell, followed by oxidation of the unsaturated carbon-carbon bond by reactive oxygen species secreted by *S. mutans*, leading to lipid peroxidation and collapsing of the bacterial cells [47]. This anti-streptococcus mutans mechanism could be similar to that of other polyunsaturated FAs such as LA.

Jung et al. investigated the anti-cariogenic properties of the Dryopteris crassirhizoma plant by using GC-MS analysis of the plant’s extract. They identified LA as the most potent antibacterial component of the n-hexane fraction against *S. mutans* bacteria. They also proved that LA had a dose dependent antibiofilm effect after a brief treatment of *S. mutans* biofilm with LA (10 minutes), which significantly reduced the biofilm dry weight. These results are in accordance with the findings of the current study [53].

*S. mutans* secrete glycosyltransferase (GTF) which is crucial for the attachment of *S. mutans* on the salivary pellicle covering the enamel surface, and for the formation of biofilm matrix. GTF forms glucans using sucrose as substrate. There are three genetically different types of GTFs, which form soluble and insoluble glucans. Glucans are adsorbed on both the bacterial cell surface and salivary pellicle, helping in the formation of biofilm [36]. Won et al. were the first to report that FAs could have anti-cariogenic effect against *S. mutans* by the inhibition of GTFs. They analyzed several herbal extracts with antibacterial effect against *S. mutans*, and found that extracts with rich content of unsaturated FAs exhibited potent GTF inhibitory effect. FAs that had one or two double bonds such as OA, showed maximum inhibition effect. They observed that unsaturated FAs such as OA and LA had higher GTF inhibition effect than saturated FAs. Extracts with OA:LA ratio of 2:1 gave the maximum GTF inhibition effect in their trials [54].

Abdel-Aziz et al. analyzed extract from the endophytic fungus ‘Arthrographis kalrae’ by GC-MS. They found that the high concentration of unsaturated FAs (OA and LA) in the fungus extract, could inhibit *S. mutans* biofilm formation on salivary coated hydroxyapatite discs, in a dose dependent manner, through inhibition of water insoluble extracellular polysaccharide formation [50].

NSE caused a significant decrease in the thickness of *S. mutans* biofilm after 10 minutes of application, in comparison to both the positive biofilm control and CHX groups. The thickness reducing effect by NSE on *S. mutans* biofilm could be related to the rich composition of LA in NSE, as stated by Jung et al., who observed a similar effect using Dryopteris crassirhizoma plant extract which is rich in LA content [53]. Accordingly, the antibiofilm effect of NSE was not related only to its antibacterial effect but might also be related to the physical action of FAs on the *S. mutans* biofilm structure. Hara et al. investigated the effect of 10 minutes of application of FAs salt on *S. mutans* biofilm formed over the surface of acrylic dentures. They concluded that unsaturated FA salts (oleate, linoleate, and linolenate) caused removal and detachment of *S. mutans* biofilm from acrylic denture surfaces by immersion, through physical action such as surfactant effect and mechanical disturbance of biofilm by osmosis induced swelling [55]. CHX has a biofilm thickness reducing effect due its cationic charge with hydrophilic and hydrophobic properties [56]. However, in the present study, NSE proved to have a higher reducing effect than CHX.

During the last decade, much attention has been drawn to the anticariogenic effect of unsaturated FAs, due to their antibacterial and antibiofilm effects against *S. mutans* [57]. Giacaman et al. found that when poly- and monounsaturated FAs (LA and OA respectively) presented to *S. mutans* biofilm on enamel specimens after cariogenic challenge, they caused a decrease in enamel demineralization, a decrease in biofilm biomass, and a decrease in insoluble extracellular polysaccharides production. They also observed that the antibiofilm effect of the unsaturated FAs was significantly higher than that of saturated stearic FA [57].

By reviewing the literature, it has been concluded that unsaturated FAs could have multiple antibacterial and antibiofilm mechanisms, which target both the *S. mutans* cell itself and the production and the stability of the extracellular polysaccharide matrix of *S. mutans* biofilm, leading to a decrease in the biofilm biomass [47, 50, 54, 55, 57]. These multiple weapons are considered a unique advantage of LA and OA, that could explain the significant decrease in *S. mutans* biofilm thickness after NSE application.

Another aim of the present study was to predict the inhibitory action of FAs in NSE against *S. mutans*. One of the possible inhibitory antibacterial mechanisms of FAs against *S. mutans*, could be targeting *S. mutans* enzymes responsible for cell wall formation, such as MurA enzyme which has a role as a catalyst during the formation of PG in the *S. mutans* cell wall [17, 36, 58]. The cell wall is responsible for preserving the shape of the bacterial cell and protecting it against osmotic pressure [59]. PG is composed of sugar polymers such as N-acetyglucosamine (NAG) and N-acetylmuramic acid, which are attached to several amino acids. MurA UDP-N-acetylglucosamine enolpyruvyl transferase enzyme catalyzes the initial step of N-acetylglucosamine-N-acetylmuramyl pentapeptide synthesis, which is considered the first step in PG biosynthesis [60]. Mammalian cells do not have homolog to MurA enzyme. Therefore inhibition of MurA enzyme is an attractive target for discovery of new bacterial-specific antibiotics [17, 59].

MD was used in the current study to explore a possible binding effect between the long chain FAs in NSE (LA, OA and palmitic acids) and active sites of MurA enzyme. The three modelled FAs showed a high binding affinity towards amino acids residues of MurA enzyme, because of the high number of formed hydrogen bonds (eight H-bonds). Especially, LA which presented the highest affinity to MuraA enzyme, followed by OA and then palmitic acid. The presence of a hydroxyl group at the end of FAs’ chains may explain the high number of formed H-bonds with the enzymatic protein. Hydrophobic bonding between the aliphatic chains of the three FAs and hydrophobic pockets of MurA enzyme was detected during docking, which added more to the strength of the affinity between FAs of NSE and MurA enzyme, and act as a driving force for conformational changes in the protein of target enzyme. Accordingly, the three long chain FAs present in NSE may have a great potential for selective inhibition of cell wall synthesis in *S. mutans*, by binding to MurA enzyme, and with expectations of a superior inhibition effect of LA in comparison to OA and palmitic acids [61].

The docking results showed that NSE could be a promising biofilm inhibitory agent against *S. mutans*. Fosfomycin is an antibiotic which specifically modifies and inactivates MurA enzyme [62]. The results of MD showed that LA, OA and palmitic acid are considered competitors to Fosfomycin, as they bonded effectively to Asn23 and Lys22 amino acids, while these two amino acid residues are also considered active sites for bonding with Fosfomycin ligand [61]. Therefore, NSE FAs may be a promising alternative to Fosfomycin, to avoid its undesirable side effects [17, 61].

According to the results of the current study, it can be concluded that NSE had an antibacterial effect against *S. mutans* biofilm comparable to that of CHX, and therefore, the first null hypothesis was accepted. NSE showed a higher biofilm removal effect than CHX, as NSE application caused a higher decrease of the biofilm thickness on the enamel specimens, and accordingly, the second null hypothesis was rejected.

NSE could be a promising alternative to CHX, as an antibiofilm agent that can be used in topical oral preparation products such as mouthwashes or oral gels, to avoid side effects of using chlorhexidine preparations such as altering taste, staining of teeth, adverse effects on oral microbiome balance [63] and the emergence of antibiotic resistance [4, 5]. Advantages of NSE may not be limited to the biofilm removal effect but they can also extend to the inhibition potential of biofilm formation, according to the results of MD.

One of the limitations of the current study was a lack of standardization of enamel specimens’ roughness, which is very important to standardize biofilm growth [56]. Also, future in vitro and in vivo studies are recommended about NSE oral applications, to evaluate its systemic and local biocompatibility, cytotoxicity, pharmacokinetics [64], effect on oral microbiome [63] and mucous membrane absorption [65, 66]. Also, the effect of NSE on biofilm formed by multiple oral bacterial and fungal species should be investigated in upcoming research [56]. Evaluation of the effect of other organic components of NSE such as glycerol or using other extraction methods of *N. sativa* seeds, should be carried out in future research studies [46].

## Conclusion

Within the limitations of this study, it can be concluded that:

1. NSE had a significant antibacterial effect against *S. mutans* biofilms, comparable to that of the commonly used chlorhexidine mouthwash. NSE had a higher potential to reduce thickness of *S. mutans* biofilm than that of CHX.
2. The rich content of long chain (C18) fatty acids may be responsible for the antibacterial and antibiofilm properties of NSE.
3. NSE is expected to have a potent inhibitory action against *S. mutans*, by inhibiting MurA enzyme action, through its rich content of long chain fatty acids. Linoleic acid is expected to have maximum inhibitory action against MurA enzyme, in comparison to other fatty acids in NSE.

## Declarations

### Availability of data and material

All data supporting the findings of the current study are available and included within the paper. Extra data about GC-MS, MD, or CLSM images are available from authors upon request.
